# Personalized Assessment for Cancer Prevention, Detection, and Treatment

**DOI:** 10.3390/ijms25158140

**Published:** 2024-07-26

**Authors:** Laura Paleari

**Affiliations:** Research, Innovation and HTA Unit, Liguria Health Authority, A.Li.Sa., 16121 Genova, Italy; laura.paleari@alisa.liguria.it

The intention of this Special Issue is to highlight research that aims to recognize cancer’s complexity to better prevent or treat its occurrence.

Personalized (or precision) medicine in oncology is grounded in the idea that cancers are not all the same and people may respond differently to treatments due to their genetic, environmental, and lifestyle characteristics [1]. Before the advent of precision medicine, cancer treatments were mainly based on standardized approaches, such as chemotherapy and radiotherapy, with significant side effects and variable results. Precision medicine has essentially changed this landscape, enabling more personalized, effective, and targeted care [1]. The improvement in the technologies used for the analysis of cancer cell DNA (the so-called NGS, next generation sequencing technologies) has led to a change in the cancer treatment paradigm, thanks to continuous studies. They are designed to address the following: identify specific genetic mutations that drive tumor growth; quantify the mutational burden, which can lead to the discovery of resistance to treatment mechanisms; and develop molecularly targeted drugs designed to inhibit specific alterations in neoplastic cells [2]. An example of personalized research is represented by Suliman and colleagues’ work, which aimed to correlate chemo-resistance with biological variables in endometrial cancer (EC) patients [3]. To reach this goal, they explored the functional association between aggressive cancer-associated fibroblast cells (CAFs), derived from EC patients, and resistance to chemo/targeted drugs [3]. Their results showed a positive correlation between grade 3 (*p* = 0.025) and stage 3/4 diseases (*p* = 0.0106) with aggressive CAFs and the post-surgery event (PSE). Furthermore, aggressive CAFs, derived from patients with PSE, displayed resistance to paclitaxel and lenvatinib [3].

Furthermore, the clinical utilization of measurable biomarkers has been possible by personalized medicine. This approach aims to avoid ineffective treatments by the evaluation of specific genetic characteristics of each patient. In the clinical setting, the Ki-67 proliferation marker is well known; it often correlates with an unfavorable prognosis in tumors [4,5], even if its use is to date has mainly been restricted to breast cancers (BCs) [6,7,8,9]. Recently, a retrospective study on a cohort of women affected by EC has been published, with the intention of exploring Ki-67’s predictive and prognostic role [9]. The results of this research suggest a positive role for the Ki-67 index as a prognostic and predictive marker of response to hormone treatments in early-stage ECs with a statistically significant benefit in terms of disease-free survival (DFS) [HR = 0.25 (95% CI; 0.09–0.69), *p* = 0.008] and overall survival (OS) [HR = 0.30 (95% CI; 0.10–0.87), *p* = 0.03] in the high-expressing Ki-67 group treated with hormone therapy [10]. Moreover, a comprehensive analysis to detect novel biomarkers has been published, with a focus on the diagnosis and progression of gastric cancer (GC) [11]. It has been demonstrated that specific chemokines and their receptors, the vascular endothelial growth factor (VEGF) and epidermal growth factor receptor (EGFR), interleukin 6 (IL-6) and C-reactive protein (CRP), matrix metalloproteinases (MMPs) and their tissue inhibitors (TIMPs), a disintegrin and metalloproteinase with thrombospondin motifs (ADAMTS), as well as DNA- and RNA-based biomarkers, and c-MET (tyrosine-protein kinase Met) play a role in the pathogenesis of GC [11].

Another important aspect of precision medicine is represented by predictive medicine, which uses the analysis of genetic characteristics to evaluate the individual risk of developing cancer or the individual response to a treatment. This can allow for early diagnosis and targeted preventive interventions, bringing patients’ life expectancy and quality of life to a higher level. For instance, taste and smell disorders (TSDs), which are common side effects in patients undergoing cancer treatments, have been studied to recognize which treatments specifically cause them with the aim to improve patients’ quality of life [12]. Buttiron Webber and colleagues identify, among cancer treatments, those that principally lead to taste and smell changes and provide evidence for wider studies, including those focusing on prevention [12]. The results of this research definitely highlight how patients’ quality of life is a crucial issue in oncology and how its advancement can be reached only with the active participation of all the professional health figures involved in cancer management.

To date, the advent of new technologies, the omics sciences, and the use of artificial intelligence are significantly contributing to the progress of precision oncology. For instance, the clinical utility of comprehensive genome profiling (CGP) tests, used in precision oncology to guide therapeutic choices, remains controversial [13,14]. Very recently, the results of a study on a learning program for treatment recommendations by molecular tumor boards (MTBs) and artificial intelligence (AI) were published [15]. The aim of this study was to examine the effectiveness of a learning program aimed at improving treatment recommendations, focusing on genomic alterations with low levels of evidence. For this purpose, simulated cases of advanced cancer were used and the efficiency of an AI-based annotation system to improve clinical decisions was examined [15]. The findings of this quality improvement study suggest that use of a learning program improved the concordance of treatment recommendations provided by MTBs compared to central ones. Treatment recommendations made by an AI system showed higher concordance than that for MTBs, indicating the potential clinical utility of the AI system.

Despite the substantial progress achieved thanks to precision medicine, it is important to underline that this field is still constantly evolving. Research studies are ongoing; nonetheless, precision medicine has already demonstrated its enormous potential in improving the effectiveness of cancer treatments and providing new hope for patients suffering from cancer.
